# An evaluation of postmarketing reports of hyperglycaemia associated with dolutegravir for treatment of HIV in Eswatini

**DOI:** 10.1186/s12981-022-00481-0

**Published:** 2022-11-24

**Authors:** Alemayehu L. Duga, Sibongile Magongo, Siphesihle Nhlabatsi, Denis O. Ladwar, Linda Härmark, Leàn Rolfes

**Affiliations:** 1 Medicine Regulatory Unit, Eswatini Ministry of Health, Matsapha, Eswatini; 2 National Pharmacovigilance Center, Eswatini Ministry of Health, Matsapha, Eswatini; 3 Baylor College of Medicine Children’s Foundation, Mbabane, Eswatini; 4 Chemonics Eswatini, Manzini, Eswatini; 5 The Netherlands Pharmacovigilance Center Lareb, s-Hertogenbosch, The Netherlands

**Keywords:** Dolutegravir, HIV/AIDS, Eswatini, Hyperglycaemia, Pharmacovigilance, Signal

## Abstract

**Background:**

Dolutegravir (DTG) is an Integrase Strand Transfer Inhibitor (INSTI) indicated in combination with other antiretroviral agents for the treatment of HIV infection. It is available in a number of pharmaceutical preparations including the fixed-dose combination (TLD) containing tenofovir (300 mg) + lamivudine (300 mg) + dolutegravir (50 mg). In 2018, Eswatini adopted TLD as the preferred first-line HIV treatment regimen for adults and adolescents as per WHO recommendations. From March 2019 to March 2020, the National Pharmacovigilance Center (NPC) in Eswatini received 8 reports of hyperglycaemia associated with the use of DTG. This study was conducted to investigate if Eswatini NPC database included cases suggestive of causality between dolutegravir and hyperglycaemia.

**Method:**

A qualitative synthesis of information from the Eswatini national pharmacovigilance database from March 2019 to March 2020 was conducted to investigate a casual association between hyperglycaemia and dolutegravir.

**Results:**

All reports with dolutegravir containing regimen and suspected Adverse Event of hyperglycaemia in the period of March 2019 to March 2020 were included in the study. Seven of the reports were serious (resulted in hospitalization and one case concerned optic neuritis, leading to blindness). Two patients had a medical history of diabetes while the rest of the patients had never experienced hyperglycaemia before starting dolutegravir. For all the reports, the time to onset of hyperglycaemia ranges from 2–5 months after the initiation of DTG. None of the patients discontinued the use of DTG. All the patients were treated with oral hypoglycaemic medication. In severe cases, patients were treated with intravenous normal saline and ringer lactate as well as rapid-acting insulins. All patients are currently stable on oral hypoglycaemic drugs.

**Conclusion:**

Cases that support causality between dolutegravir containing regimen and hyperglycaemia were found. These cases were mainly serious. Based on these findings it is recommended that healthcare professionals (HCPs) actively screen all patients for risk factors of hyperglycaemia before DTG initiation. In addition, it is important that HCPs are aware of the possible association between DTG and hyperglycaemia.

## Background

Since the approval of zidovudine (AZT) in 1987, more than 25 antiretroviral agents in six classes have been approved to treat HIV infection [1]. These include nucleoside reverse transcriptase inhibitors (NRTIs), nucleotide reverse transcriptase inhibitors (NtRTIs), non-nucleoside reverse transcriptase inhibitors (NNRTIs), protease inhibitors (PIs), fusion inhibitors (FIs), and integrase inhibitors (INSTIs) [2]. Dolutegravir is a new INSTI approved for combination treatment in HIV infected patients [3]. In 2013, the U.S. Food and Drug Administration approved dolutegravir **(**Tivicay**®)** tablets, in combination with other antiretroviral agents for the treatment of HIV infection in adults and children aged 12 years and older and weighing at least 40 kg [4]. It was also added to the 20th edition of the World Health Organization (WHO) Essential Medicine List in 2017 [5]. It is available as mono and fixed-dose combination (TLD), together with tenofovir (TDF) and lamivudine (3TC).

TDF belongs to a class of antiretroviral drugs known as nucleotide analogue reverse transcriptase inhibitors (NtRTIs), which block reverse transcriptase, an enzyme necessary for viral production in HIV-infected individuals [6]. 3TC is a nucleoside analogue which is incorporated into viral DNA by HIV reverse transcriptase and HBV polymerase, resulting in DNA chain termination, while DTG inhibits HIV integrase by binding to the active site and block the strand transfer step of retroviral DNA integration in the host cell [7, 8].

In Eswatini the first-line treatment for HIV is a fixed-dose combination (TLD), which contains TDF 300 mg + 3TC 300 mg + DTG 50 mg. The fixed-dose combination is available in the form of tablets for adults and adolescents. Due to DTG’s high viral suppression abilities, newer paediatric formulations containing 25 mg and 10 mg DTG are being developed and are due to become available in the latter half of 2021[9].

The Eswatini National AIDS Programme adopted the TLD regimen as the preferred first-line HIV treatment regimen for adults and adolescents in the country as per WHO recommendations in 2018 [10, 11]. This policy change initiated a transition of approximately 80% of the over 191,000 people living with HIV (PLHIV) on efavirenz (EFV) and nevirapine (NVP) based regimens to DTG based regimens in the period October 2018 to March 2020. Currently, over 68% of PLHIV are on TLD and other DTG containing regimens [12].

Known adverse drug reactions (ADRs) in relation to DTG are insomnia, nausea, diarrhoea, dizziness, and headache [11, 13]. The National Pharmacovigilance Center (NPC) in Eswatini received 8 reports of hyperglycaemia associated with the use of DTG from March 2019 to March 2020. Hyperglycaemia can cause severe implications for the patient. Timely recognition and treatment is necessary. This case series assessment aims to describe the cases and the possible relation between treatment with DTG and the occurrence of hyperglycaemia in Eswatini.

## Methods

Pharmacovigilance reports were identified from the Eswatini national pharmacovigilance database from March 2019 (introduction of DTG) to March 2020. All reports with DTG containing regimen and suspected AE of hyperglycaemia in the period of March 2019 to March 2020 were included in the study. In addition, Africa (continental) and global cases were extracted from VigiBase, the WHO global database of individual case safety reports, using MedDRA [14] preferred terms (Hyperglycaemia/new onset Diabetes mellitus and Diabetes) as a search criteria) which were used to support the in-country data. A qualitative descriptive analysis was then performed on the eligible reports from the database. The reports were then stratified by sex (male, female), age and seriousness (serious and non-serious). Excel were utilized for data cleaning and data analysis.

## Results

The NPC analysed in-country reports in relation to DTG-containing regimen submitted in the period of March 2019 to March 2020. The Center received a total of 115 reports on DTG of which 8 reports were in relation hyperglycaemia. In global view, 961 reports out of 10,600 total reports on DTG containing regimens were hyperglycaemic cases; similarly 272 reports out of 1,233 reported in Africa are DTG-hyperglycaemia associated cases Hyperglycaemia is blood glucose greater than 7 mmol/l while fasting and greater than 10 mmol/l two hours postprandial [15]. All the patients included in the report are above 50 years of age, 6 were females and 2 males. None of the patients were discontinued from the DTG containing treatment nor the dose of DTG was changed and all patients were recovering from the hyperglycaemia after treatment with anti-diabetic treatment.

Seven of the cases were serious with six resulted in hospitalizations and one case of optic neuritis leading to blindness. Out of the 8 cases, 2 cases had a medical history of diabetes while the rest of the reports were newly diagnosed. For all the reports, the time to onset of the reaction ranges from 2–5 months after the initiation of the treatment. All of the patients were treated with oral hypoglycaemic medication to lower the blood glucose level. In severe cases, patients were treated with IV normal saline, ringer lactate as well as rapid-acting insulins. All patients are currently stable on hypoglycaemic drugs. Table 1 shows the eight hyperglycaemic cases attributed to DTG and DTG containing regimen.

**Table 1:** List of hyperglycaemic cases

Sex and age	a.Suspected drug b.Strength c.Frequency of administration d.Indication	Treatment of ADR	Concomitant drug	ADR reported	Serious and seriousness criteria	Time to onset	Outcome of the ADR
Female, 51	a.Dolutegravir; Lamivudine; Tenofovir b.50 mg/300 mg/300 mg c.Once a day d.HIV infection	Metformin 500 mg	Isoniazid	Hyperglycaemia	Yes; hospitalization	5 months	Recovering
Female, 52	a.Dolutegravir; Lamivudine; Tenofovir b.50/300 mg/300 mg c.Once day d.HIV infection	Metformin 500 mg and Glibenclamide 5 mg		Hyperglycaemia	Yes; hospitalization	2 months	Recovering
Male, 64	a.Abacavir; Dolutegravir; Lamivudine b.Once a day c.HIV infection	1. Insulin and normal saline 2.Metformin 500 mg		Hyperglycaemia Blindness	Yes; hospitalization	4 months	Recovering
Female, 52	a.Dolutegravir; Lamivudine; Tenofovir b.50 mg/300 mg/300 mg c.Once a day d.HIV infection	Continued with Metformin and Glibenclamide	Metformin Glibenclamide	Hyperglycaemia Muscle spasms Dizziness	Yes; hospitalization	3 months	Recovering
Female, 57	a.Dolutegravir; Lamivudine; Tenofovir b.50 mg/300 mg/300 mg c.Once a day d.HIV infection	Metformin 500 mg	Hydrochlorothiazide Acetylsalicylic acid	Hyperglycaemia	Yes; hospitalization	4 months	Recovering
Female,70	a.Dolutegravir; Lamivudine; Tenofovir b.50 mg/300 mg/300 mg c.Once a day d.HIV infection	Metformin 500 mg	Atenolol Hydrochlorothiazide	Hyperglycaemia Muscle spasms	Yes; hospitalization	3 months	Recovering
Male, 58	a.Dolutegravir; Lamivudine; Tenofovir b.50 mg/300 mg/300 mg c.Once a day d.HIV infection	Continued with Metformin 500 mg	Metformin 500 mg	Hyperglycaemia Muscle spasms	No	3 months	Recovering
Female	a.Dolutegravir; Lamivudine; Tenofovir b.50 mg/300 mg/300 mg c.Once a day d.HIV infection	•Insulin 12 IU •Infused with ringer lactate solution •Metformin 500 mg •Glibenclamide 5 mg	Hydrochlorothiazide Enalapril	Hyperglycaemia	Yes; hospitalization	5 months	Recovering

## Discussion

As with many new medicines, post market surveillance of DTG on a large scale is very important to establish its full safety and side effect profile. This case series contributes to the knowledge about the possible association of hyperglycaemia and DTG treatment. Known factors contributing to hyperglycaemia include reduced insulin secretion, decreased glucose utilization, and increased glucose production, and several medicine, like antihypertensive drugs and protease inhibitors [15]. Additionally, factors like advancing age, male gender, longer duration of HIV infection, low CD4 count, high viral burden, high body mass index, obesity, lower socioeconomic class, comorbidity and drugs should be taking into consideration as contributing factors for hyperglycaemia when it occurs in patient on DTG [16].

A few clinical trial studies and review papers strengthen the evidence that integrase inhibitors (the drug class that DTG belongs to) can induce hyperglycemia [17, 18]. According to VIKING-3, a single armed phase III clinical trial, hyperglycaemia was one of the most common laboratory abnormalities in 14% of the patients at week 48 of integrase strand transfer inhibitor therapy [17].

A case–control study on the incidence of DTG-associated hyperglycaemia in patients with HIV from Uganda also indicates that patients who were transitioned to DTG-based first-line regimens had a higher incidence (16 (0.47%) of 3417 patients) of hyperglycaemia than patients who did not transitioned to DTG 1(0.03%) of 3230 patients in the control group (p = 0.0004) [18]. In literature, there are also a few case reports that support this association. For all the reports, the time to onset of the reaction ranges from 2–5 months after the initiation of the treatment. that has similarities with a study conducted in Uganda which indicates the mean onset of the ADR as 4 months [18]. In a case report, a patient presented to the emergency department (ED) with hyperglycaemia approximately 3 weeks after the switch from EFV to DTG [19].

In contrast to the above findings, the investigation from 4 DTG clinical trials (SPRING-1, STRIIVING, SWORD-1 and -2) on the potential effect of DTG on insulin resistance over time found no association between treatment and insulin resistance observed over a 48 week period [20].

A mechanism for the DTG induced hyperglycaemia is not well understood. However, chelation of magnesium that inhibits the release and signalling of insulin was hypothesized as a mechanism of the toxicity [19]. According to the update of recommendations on first- and second-line antiretroviral regimens issued by the WHO, there is between 3 and 5 kg of unintentional weight gain in individuals receiving DTG-based regimens at 48 weeks, however nothing has been mentioned about hyperglycaemia in relation to the treatment in the update [21]. Hyperglycaemia and weight gain related to DTG are published in the FDA Product information based on clinical trial and post marketing surveillance studies [22]. However, the ADRs are not listed in the Summary of Product Characteristics (SmPC) of the European Medicines Agency (EMA) [23].

The NPC has not received hyperglycaemic cases associated to other HIV medicines during the review period. The SmPC of lamivudine does however include the occurrence of increase in weight and in levels of blood lipids and glucose [23]. Another study on a cohort of 533 HIV-infected and 755 HIV-seronegative men in the Multicentre AIDS Cohort Study evaluated indicates that insulin resistance was seen more commonly among patients with longer exposure to NRTI, in older patients, and in non-white patients (24). Therefore, longer NRTI exposure could have an additive effect to the DTG hyperglycaemic property. The findings of this research will create an opportunity to improve future research on this area and contribute further understanding on DTG-hyperglycaemia association.

## Limitation of the study

There could be under reporting of the cases as the case series study was analysed from the ADR reports reported to the pharmacovigilance center, which may not reflect the true picture of the hyperglycaemia cases caused by DTG in the whole country.

## Conclusion

This case series contributes to knowledge about the possible association between the use of DTG and hyperglycaemia. Based on the cases received by the NPC and supporting information from VigiBase and literature, we consider hyperglycaemia to be a potential side effect of DTG. The hyperglycaemia can however successfully be treated with oral antidiabetic drugs while continuing DTG treatment. We would also like to emphasize that the necessity of awareness creation for health care workers and patients on the timely recognition of hyperglycaemic cases while on this treatment. Close monitoring of serum plasma glucose should be considered after the initiation of an INSTI.

## Data Availability

Data belong to the Kingdom of Eswatini Ministry of Health (MoH). The MoH reserves the right to review and approve of all public health data before it is released to the public. Therefore, the authors of this manuscript do not have the right to make all the data underlying the manuscript fully available and without restrictions. Permission to access data needs to be requested in writing from the MoH.

## Abbreviations

3TC: Lamivudine
ABC: Abacavir
DTG: Dolutegravir
EFV: Efavirenz
EMA: European medicine agency
HBV: Hepatitis B virus
ICSRs: Individual case safety reports
LPV/r: Lopinavir/Ritonavir
NPVU: National pharmacovigilance unit
NVP: Nevirapine
PLHIV: People living with HIV
RBS: Random blood sugar
TDF: Tenofovir
TLD: Tenofovir, Lamivudine and Dolutegravir
